# Prediction of active marker of seabuckthorn polysaccharides for prevention and treatment of cervical cancer and mechanism study

**DOI:** 10.3389/fnut.2023.1136590

**Published:** 2023-02-09

**Authors:** Xuehua Feng, Zurong Song, Ali Tao, Panpan Gong, Wenqing Pei, Rumin Zong

**Affiliations:** College of Pharmacy, Anhui Xinhua University, Hefei, Anhui, China

**Keywords:** seabuckthorn polysaccharides, cervical cancer, network pharmacology, target point, mechanism of action

## Abstract

**Objective:**

To predict the target of Seabuckthorn polysaccharides in the prevention and treatment of cervical cancer, and to explore its multi-target and multi-pathway mechanism.

**Methods:**

Using the Swisstarget database, a total of 61 potential targets of polysaccharide active components were obtained. Cervical cancer related targets were obtained from the GeneCards database. The correlation score was greater than 5 targets for 2727; 15 intersection targets of active ingredients and disease were obtained by Venn diagram. Cytoscape3.6.0 software was used to construct the Polysaccharide composition-Target-Disease Network and Protein-Protein Interaction Networks (PPI). Cytoscape3.6.0 software was used for visualization and network topology analysis to obtain core targets. Kyoto encyclopedia of Genes and Genomes (KEGG) and Gene Ontology (GO) were analyzed using Metascape database. SailVina and PyMOL software were used for molecular docking to verify binding strength.

**Results:**

A total of 15 core targets were obtained for cervical cancer. These targets are significantly enriched in HIF-1 signaling pathway, Galactose metabolism, EGFR tyrosine kinase inhibitor resistance, growth factor receptor binding, carbohydrate binding, protein homodimerization activity and other GO and KEGG entries; Molecular docking showed that ADA and GLB1 were well bound to Glucose, D-Mannose, and Galactose.

**Conclusion:**

The effect of seabuckthorn polysaccharides on the prevention and treatment of cervical cancer is characterized by multi-component, multi-target and multi-pathway, which provides scientific basis for further research on the activity of seabuckthorn polysaccharides.

## 1. Introduction

The normal cells in the tumor organism have undergone qualitative changes under the long-term action of numerous internal causes (such as heredity, endocrine disorders, malnutrition and other conditions, tension, etc.) and external causes (such as physical, chemical, biological, etc.), thus having the ability of excessive proliferation. Malignant tumors are called cancers, cervical cancer is a type of cervical malignancy with the highest incidence among gynecological malignancies, cervical cancer is the most common cause of cancer-related deaths in women in 42 low-income and lower-middle-income countries (1, 2). In recent years, its incidence tends to be younger and younger, which is a serious threat to women’s health. Cervical cancer is characterized by late detection, high degree of malignancy, poor prognosis and imprecise treatment (3). Modern medical treatment of cervical cancer mainly involves surgical resection and chemoradiotherapy. However, the side effects of chemoradiotherapy are relatively large, and the body immunity of post-operative patients is reduced. Traditional Chinese medicine plays an ideal role in the adjuvant treatment of cervical cancer, which can improve the clinical symptoms of patients with cervical cancer, improve their immunity, and further improve their quality of life (4).

Carbohydrates are mainly monosaccharides, disaccharides, polysaccharides and other types according to their structure. Monosaccharides are the simplest sugars. In general, human digestive enzymes will not break them down into smaller sugars. Polysaccharides are macromolecular compounds that are polymerized from more than 10 monosaccharide molecules. Its characteristics are very different from monosaccharides, disaccharides and oligosaccharides, etc. Polysugars are generally insoluble in water, sweet, can’t form crystallization, no reducing property (5). Seabuckthorn contains a variety of active ingredients, such as polysaccharide, flavonoids, tannins, terpenoids and vitamins, which have extensive pharmacological activities such as anti-tumor, prevention of cardiovascular and cerebrovascular diseases, anti-inflammation, anti-oxidation, liver protection and bacteriostasis (6). Polysaccharide is a kind of natural polymer, non-toxic, with few side effects and wide biological activity. It is a naturally occurring repeating unit (monosaccharide or oligosaccharide) polymerized carbohydrate, which is linked together by glycosidic bond and is an essential biological macromolecule in life activities. It has functions of immune regulation, anti-tumor and anti-oxidation, etc., and has been widely studied in functional and medical fields (7–9). As a kind of compound with high antitumor activity, natural polysaccharide has been widely concerned. Compared with traditional chemotherapy drugs, natural polysaccharide shows low toxicity or non-toxicity to normal cells, and is more biosafe. In China, seabuckthorn resources are very rich, so for the development of drugs and functional food, seabuckthorn is a very considerable source, so it is particularly important to study its anti-tumor mechanism.

Network pharmacology is a branch of pharmacology of bioinformatics and high-throughput omics analysis. It can be used to study the mechanism of action of TCM prescriptions and monomers on diseases. In this study, network pharmacology was adopted to study the multi-target and multi-pathway action characteristics of seabuckthorn polysaccharide, and to explore the active substance basis and action mechanism of seabuckthorn polysaccharide in the prevention and treatment of cervical cancer.

## 2. Analysis method

### 2.1. Collection and target prediction of active chemical constituents of seabuckthorn polysaccharides

Seabuckthorn contains flavonoids, tannins, terpenoids, polysaccharides, vitamins and other active components. Flavonoids are the main functional compounds of seabuckthorn. At present, more than 50 kinds of flavonoids have been isolated and identified from seabuckthorn plants, mainly using isorhamnetin, quercetin, and kaempferol as aglycones, which are combined with glucose, rhamnose, arabinose and rutin to form glycosides. There are more than 20 triterpenoids in sea-buckthorn, mainly including oleanolic acid, ursolic acid, corosolic acid and other triterpenoids (10). Polysaccharide refers to biological macromolecules composed of ketoses or aldoses connected by glycosidic bonds, polysaccharide is formed by condensation of several monosaccharide molecules and loss of water. When polysaccharide enters the human body, it must be hydrolyzed into monosaccharide catalyzed by enzymes before it can be absorbed. In seabuckthorn, the main active ingredient is seabuckthorn polysaccharides, which has important research value and has been extensively studied in recent years. The literature showed that (11) the polysaccharides isolated from seabuckthorn were mainly neutral polysaccharides composed of glucose, fructose, galactose, arabinose, mannose and rhamnose, among which glucose and fructose accounted for 80% of the total sugars, and the polysaccharide content of seabuckthorn fruit was higher than that of seabuckthorn leaves. In this study, the compound information of seabuckthorn polysaccharides was obtained by using PubChem database, and the obtained active ingredients were input into Swisstarget to obtain the corresponding target of each active ingredient.

### 2.2. Cervical cancer related target retrieval

“Cervical Cancer” as keywords retrieval Gene Cards database, ^1^ screening Cervical Cancer related targets (12). Venn diagram was drawn to obtain the common target of seabuckthorn polysaccharides and cervical cancer, that is, the potential target of seabuckthorn polysaccharides on cervical cancer.

### 2.3. Construction of “seabuckthorn polysaccharides–Target-cervical cancer” network

The intersection targets of seabuckthorn polysaccharides and cervical cancer obtained from the above screening were taken as nodes, and the interactions between nodes were taken as edges. The process of seabuckthorn polysaccharides acting on cervical cancer was visualized and analyzed by using Cytoscape 3.6.0 software to build the network diagram (13).

### 2.4. Construction of protein-protein interaction Networks (PPI)

Enter the target of polysaccharide screening on cervical cancer in the STRING database, ^2^ and set the minimum interaction threshold as “high confidence” (>0.4), limited the study species as “homo sapiens,” obtained the PPI network, obtained the protein interaction information, and utilized Cytoscape 3.9.0 software to draw the PPI network, and screened out the core targets according to the node degree (14).

### 2.5. Target enrichment analysis and visualization

The intersection targets of seabuckthorn polysaccharides in the treatment of cervical cancer were imported into the Metascape database, and the species “homo sapiens” was selected for Gene Ontology (GO) enrichment analysis and Kyoto encyclopedia of Genes and Genomes (KEGG) pathway enrichment analysis. According to the *P*-value, the top items are selected for analysis (15).

### 2.6. Molecular docking

When constructing PPI network, has a high value in the process of topology analysis of seabuckthorn polysaccharides core active ingredients, and obtain the phase should be the key targets, the PubChem database ^3^ to lose screening of the main active ingredient, download a small molecule ligands 3D structure. Input the screened core target proteins in the PDB ^4^ database, download the 3D structure of the target proteins, prepare the ligand files and receptor files, use SailVina and PyMOL software for molecular docking verification, the smaller the docking fraction, the stronger affinity. A docking fraction less than −5.0 indicates a strong affinity between the docking compound and the target (16).

## 3. Results and analysis

### 3.1. Main active components of seabuckthorn polysaccharides

According to literature (11), the chemical composition of seabuckthorn polysaccharides consists of Glucose, Fructose, Galactose, etc. Based on PubChem database, the chemical structure of the active ingredient of seabuckthorn polysaccharides was confirmed, and the corresponding active ingredient was obtained from the database. The specific compound information is shown in Table 1.

**TABLE 1:** Information of seabuckthorn polysaccharides.

No	Name	PubChem ID	Smile
1	Glucose	5793	C(C1C(C(C(C(O1)O)O)O)O)O
2	Fructose	5984	C(C(C(C(C(= O)CO)O)O)O)O
3	Galactose	6036	C(C1C(C(C(C(O1)O)O)O)O)O
4	L-arabinose	439195	C1C(C(C(C(O1)O)O)O)O
5	D-Mannose	18950	C(C1C(C(C(C(O1)O)O)O)O)O
6	L-Rhamnose	439710	CC1C(C(C(C(O1)O)O)O)O

### 3.2. Acquisition of the intersection target of seabuckthorn polysaccharides and cervical cancer

**FIGURE 1:**
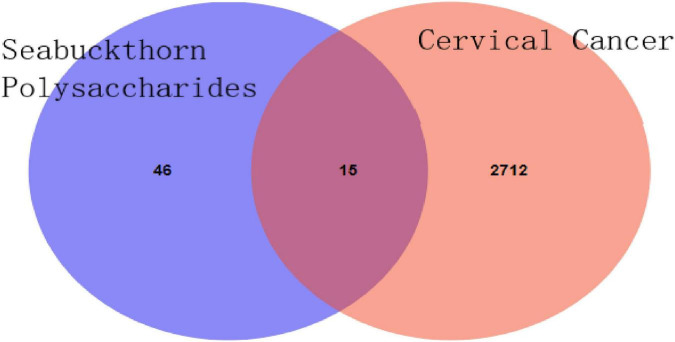
Venn diagram of seabuckthorn polysaccharides and cervical cancer targets.

The SMILE number of polysaccharide components was input into the Swisstarget database, and the species was selected as “homo sapiens,” thus the potential target could be obtained (17). The Swisstarget database was retrieved, and a total of 203 targets were obtained. The same targets were de-processed, and 61 targets were finally selected, mainly including ADORA1, ADORA2A, Adora3, ADA, etc. “Cervical Cancer” as keywords retrieval Gene Cards (see text footnote 1) database, access to Cervical Cancer related target genes, 7819, A total of 2,727 cervical cancer-related disease targets with correlation score greater than 5 were screened, and the Venn diagram of the target of seabuckthorn polysaccharides and the target of cervical cancer was drawn. The results were shown in Figure 1, and 15 core anti-cervical cancer targets of seabuckthorn polysaccharides were obtained. The results showed that seabuckthorn polysaccharides acted on the above 15 related targets in the treatment of cervical cancer.

### 3.3. Construction of “seabuckthorn polysaccharides–Target–Cervical cancer” network

**FIGURE 2:**
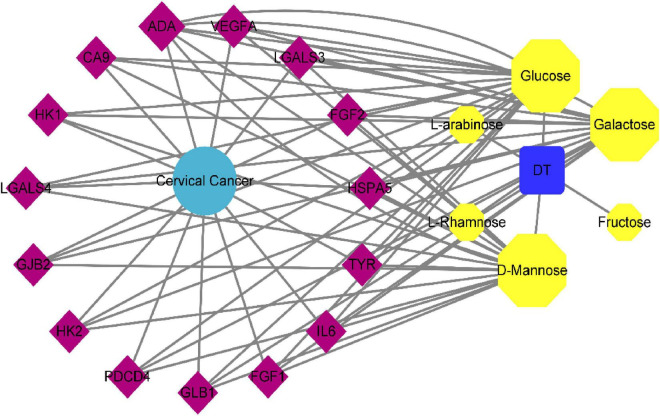
Network diagram of “Seabuckthorn polysaccharides–target–cervical cancer” interaction.

The “polysaccharides target-cervical cancer” network of seabuckthorn was constructed by Cytoscape 3.6.0 software, and the results were shown in Figure 2. In the figure, the purple quadrilateral nodes represent the target, the yellow hexagonal nodes represent the active components of seabuckthorn polysaccharides, the sky blue circle represents the disease, and the blue square represents seabuckthorn polysaccharides.

In the network, the more links between the gene target and the compound node, the bigger the shape, which means that the node is more important in the whole network, and the size of the point is expressed by the degree. The larger the degree, the closer the relationship between the target and the compound, the more likely it is to be the key target of the compound (18). The results of network topology analysis showed that active ingredients had a lot of interaction with many targets, and the top 3 active ingredients with a degree were Glucose, D-Mannose, and Galactose, indicating that these active ingredients were the main active ingredients in the treatment of seabuckthorn polysaccharides for cervical cancer. The top 5 targets were ADA, GLB1, HSPA5, CA9, and GJB2, which fully indicated that seabuckthorn polysaccharides played a role in the prevention and treatment of cervical cancer through multi-target and multi-pathway synergistic mechanism.

### 3.4. PPI network construction and topology analysis

**FIGURE 3:**
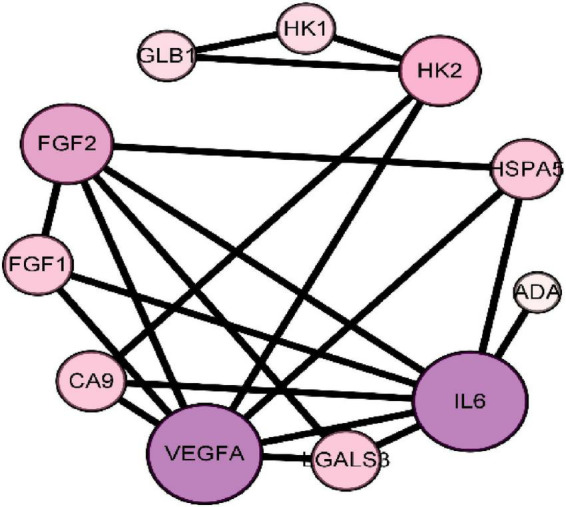
Protein-protein interaction PPI network of core target of seabuckthorn polysaccharides for prevention and treatment of cervical cancer.

Protein-Protein Interaction Networks is composed of proteins that interact with each other to participate in various aspects of the life process, such as biological signal transmission, gene expression regulation, energy and substance metabolism, and cell cycle regulation (19). Through systematic analysis of the interaction between a large number of proteins in the biological system, PPI network diagram can be used to understand the working principle of proteins in the biological system, the reaction mechanism of biological signals and energy and substance metabolism in special physiological states such as diseases, and the functional relationship between proteins (20). The 15 intersection targets were imported into the STRING database to obtain the PPI relationship. The results are shown in Figure 3. Figure 3 shows that there are 11 nodes in the network. The ranking according to the degree is shown in Table 2.

**TABLE 2:** Information on key targets of seabuckthorn polysaccharides for prevention and treatment of cervical cancer PPI network.

No	Target	Degree
1	IL6	7
2	VEGFA	7
3	FGF2	5
4	HK2	4
5	CA9	3
6	FGF1	3
7	LGALS3	3
8	HSPA5	3
9	GLB1	2
10	HK1	2
11	ADA	1

The results of network topology analysis showed that the targets of the anti-cervical cancer effect of seabuckthorn polysaccharides interacted and acted synergistically. The size and color of a node indicate the size of the degree. The larger the node, the greater the degree. The color from lavender to purple, the greater the degree. It is generally believed that the larger the degree is, the more important the target is and it is the core target of the network. Therefore, the targets IL6, VEGFA, and FGF2 are the core targets of the protein interaction network.

### 3.5. Enrichment analysis of target function and pathway

**FIGURE 4:**
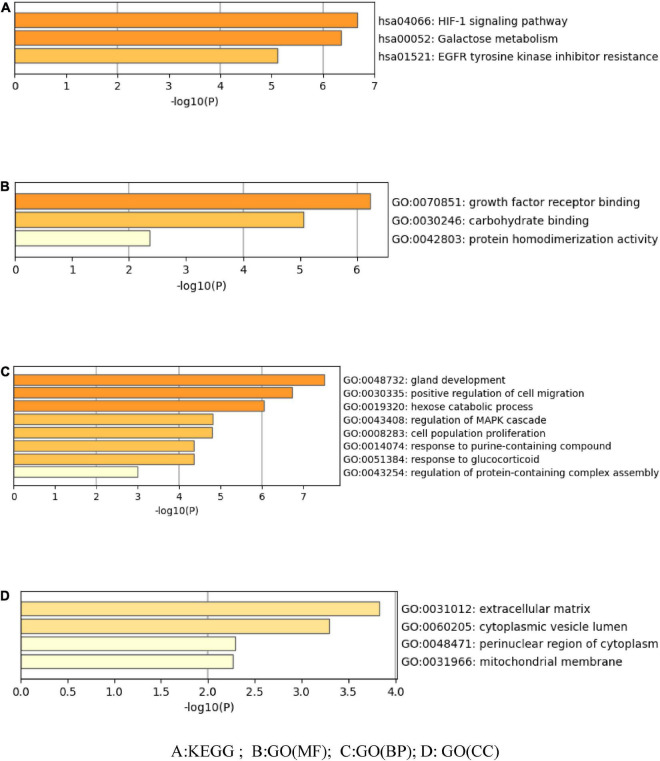
Enrichment analysis of potential targets of seabuckthorn polysaccharides. **(A)** KEGG; **(B)** GO (MF); **(C)** GO (BP); **(D)** GO (CC).

The Metascape data platform was used to analyze the signal pathway of seabuckthorn polysaccharides in the treatment of cervical cancer. The results show that the function of multiple targets is closely related to the generation of cervical cancer. The main pathways involved in seabuckthorn polysaccharides are HIF-1 signaling pathway, Galactose metabolism, EGFR tyrosine kinase inhibitor resistance, as shown in Figure 4A. The functions of relevant targets in the treatment of cervical cancer are mainly concentrated in growth factor receptor binding, carbohydrate binding, protein homodimerization activity, etc., as shown in Figure 4B. Biological processes (BP) involved include gland development, positive regulation of cell migration, hexose catabolic process, regulation of MAPK cascade, etc., as shown in Figure 4C. Cellular Component (CC) includes extracellular matrix, cytoplasmic vesicle lumen, perinuclear region of cytoplasm, mitochondrial membrane, see Figure 4D. The target pathway enrichment data information is shown in Table 3.

**TABLE 3:** Target pathway enrichment analysis results of seabuckthorn polysaccharides for prevention and treatment of cervical cancer.

GO	Description	Log10(P)	Symbols
hsa04066	HIF-1 signaling pathway	−6.67080046	HK1, HK2, IL6, VEGFA
hsa00052	Galactose metabolism	−6.353376078	GLB1, HK1, HK2
hsa01521	EGFR tyrosine kinase inhibitor resistance	−5.11426706	FGF2, IL6, VEGFA, FGF1, PDCD4
hsa04151	PI3K-Akt signaling pathway	−4.638865891	FGF1, FGF2, IL6, VEGFA
hsa05200	Pathways in cancer	−3.954612002	FGF1, FGF2, IL6, VEGFA
hsa05167	Kaposi sarcoma-associated herpesvirus infection	−3.948734921	FGF2, IL6, VEGFA
hsa05205	Proteoglycans in cancer	−3.877942402	FGF2, VEGFA, PDCD4
hsa04015	Rap1 signaling pathway	−3.847042348	FGF1, FGF2, VEGFA
hsa04014	Ras signaling pathway	−3.703075894	FGF1, FGF2, VEGFA
hsa04020	Calcium signaling pathway	−3.676178348	FGF1, FGF2, VEGFA
hsa04010	MAPK signaling pathway	−3.41777614	FGF1, FGF2, VEGFA
GO:0048732	Gland development	−7.51233	ADA, FGF2, HK2, IL6, TYR, VEGFA, CA9, LGALS3
GO:0030335	Positive regulation of cell migration	−6.73502	FGF1, FGF2, HSPA5, IL6, LGALS3, VEGFA, HK2, ADA, PDCD4, CA9, GJB2
GO:0019320	Hexose catabolic process	−6.04624	GLB1, HK1, HK2, HSPA5, ADA, IL6, LGALS3
GO:0043408	Regulation of MAPK cascade	−4.80284	FGF1, FGF2, IL6, VEGFA, PDCD4, HSPA5, GJB2, LGALS4, ADA
GO:0008283	Cell population proliferation	−4.79081	ADA, FGF1, FGF2, IL6, TYR, GLB1
GO:0014074	Response to purine-containing compound	−4.35101	ADA, HSPA5, TYR, IL6
GO:0051384	Response to glucocorticoid	−4.35101	GJB2, GLB1, IL6, CA9
GO:0043254	Regulation of protein-containing complex assembly	−2.99827	HSPA5, LGALS3, VEGFA, PDCD4
GO:0070851	Growth factor receptor binding	−6.23453	FGF1, FGF2, IL6, VEGFA, LGALS3, HK1, HSPA5
GO:0030246	Carbohydrate binding	−5.06962	HK1, HK2, LGALS3, LGALS4
GO:0042803	Protein homodimerization activity	−2.35342	GLB1, TYR, VEGFA
GO:0031012	Extracellular matrix	−3.82738	FGF1, LGALS3, LGALS4, VEGFA
GO:0060205	Cytoplasmic vesicle lumen	−3.29077	ADA, GLB1, VEGFA, TYR
GO:0048471	Perinuclear region of cytoplasm	−2.28852	GJB2, GLB1, TYR
GO:0031966	Mitochondrial membrane	−2.2639	HK1, HK2, LGALS3

### 3.6. Molecular docking

**FIGURE 5:**
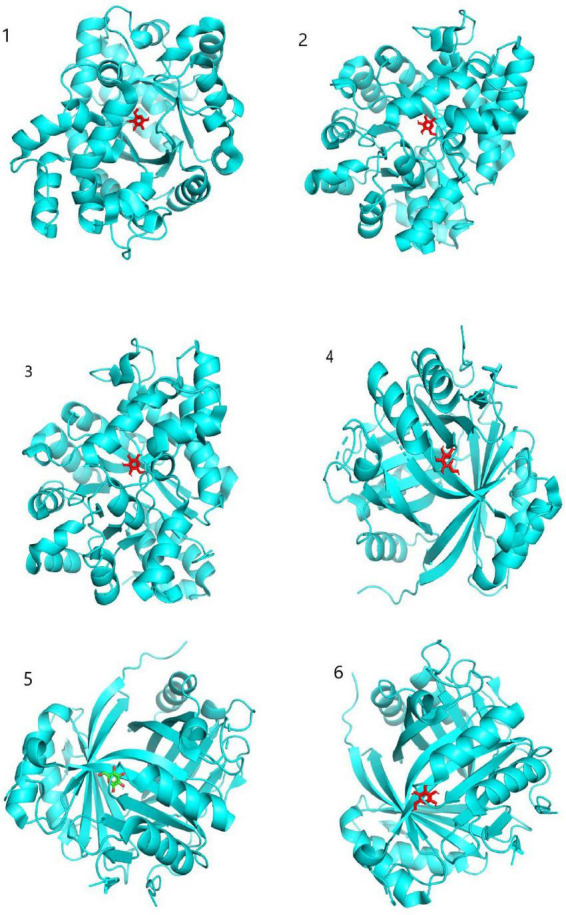
Molecular docking model diagram. 1: Glucose connects to ADA; 2: galactose connects to ADA; 3: D-Mannose connects to ADA; 4: glucose connects to GLB1; 5: galactose connects to GLB1; 6: D-Mannose connects to GLB1.

Target proteins ADA, GLB1, and HSPA5, which ranked high in the middle value of “seabuckthorn polysaccharides-target-cervical cancer” network, were selected, combined with active ingredients Glucose, D-Mannose, and Galactose, which ranked high in the middle value of network. The three-dimensional structure of active ingredient interaction was searched through PubChem database. Download the structural formula of compound MOL and use SailVina and PyMOL software for molecular docking. The results are shown in Table 4. A larger absolute value indicates that the binding force between a compound and the target is stronger, the affinity between the receptor and ligand is higher, the docking activity is greater, and the possibility of interaction is greater (21, 22). Molecular docking results showed that ADA and GLB1 had strong binding ability to Glucose, D-Mannose, and Galactose. As shown in Figure 5, the specific sites where the active ingredient binds to the target protein can be observed.

**TABLE 4:** Molecular docking results of key active ingredients and core targets.

Target	Chemical composition	Binding energy (kcal/mol)
ADA	Glucose	−6.6
ADA	Galactose	−6.7
ADA	D-Mannose	−6.7
GLB1	Glucose	−6.5
GLB1	Galactose	−6.7
GLB1	D-Mannose	−6.7

## 4. Discussion

Cervical cancer is a kind of malignant tumor that seriously threatens the life and health of women all over the world. Combined with the treatment concept of traditional Chinese medicine, traditional Chinese medicine treatment often has more advantages in improving the quality of life of patients, alleviating the symptoms of patients, controlling the spread of lesions, preventing recurrence after recovery and prolonging the survival period (23–26).

In this study, the mechanism of seabuckthorn polysaccharides against cervical cancer was investigated by means of network pharmacology. A total of 15 core targets related to the action of Seabuckthorn polysaccharide on cervical cancer were obtained, based on the “polysaccharide composition-target-cervical cancer” network, Glucose, D-Mannose, and Galactose were the active bases for seabuckthorn polysaccharides prevention and treatment of cervical cancer. The PPI network showed that IL6, VEGFA, and FGF2 are the key targets of seabuckthorn polysaccharides in the prevention and treatment of cervical cancer. GO enrichment analysis showed that BP involved included gland development, positive regulation of cell migration, hexose catabolic process, regulation of MAPK cascade, etc. KEGG pathway enrichment analysis showed that the main pathways involved in seabuckthorn polysaccharides were HIF-1 signaling pathway, Galactose metabolism, EGFR tyrosine kinase inhibitor resistance, Multiple signaling pathways play a role in preventing cervical cancer. The results of molecular docking showed that ADA and GLB1 were well combined with Glucose, D-Mannose, and Galactose, which provided a basis for prediction. Bao Xiaowei et al. (27) found that seabuckthorn polysaccharides can regulate the growth, apoptosis, metastasis and invasion of Hep-G2 cells to a certain extent, reduce the expression of MMP-2 and MMP-9 proteins by down-regulating the activity of p38 MAPK pathway, and then inhibit the migration and invasion ability of Hep-G2 cells, suggesting certain anti-tumor effects. Song Yan et al. (28) found that polysaccharides from Taxus cuspidate down-regulated the expressions of Survivin, B-cell lymphoma-2 gene (Bcl-2) and Caspase-3, thus promoting the apoptosis of cervical cancer cells. Jiang Hongwei (29) analyzed the effects of FIG leaf polysaccharide FCPS on lymphocyte proliferation and serum levels of TNF-α and IL-1β, and found that serum levels of TNF-α and IL-1β were significantly increased, and FCPS could effectively enhance the secretion of TNF-α and IL-1β in mice. It can induce cell chemotaxis and surface adhesion molecule expression, and play the role of anti-epidemic regulation to kill cervical cancer cells indirectly. Xu et al. (30) isolated and purified a new polysaccharide ACPS-1 with a molecular weight of 11.2 kDa from atractylodes, and found that ACPS-1 could significantly inhibit the proliferation of cervical cancer HeLa cells. A large number of studies have shown that natural plant polysaccharide has a good effect on inhibiting the proliferation of cervical cancer cells.

This study preliminarily explained the mechanism of action of seabuckthorn polysaccharides in the prevention and treatment of cervical cancer, and provided a reference for further *in vitro* and *in vivo* experimental verification of seabuckthorn polysaccharides in the prevention and treatment of cervical cancer and screening and evaluation of anti-cancer traditional Chinese medicine.

## Data availability statement

The datasets presented in this study can be found in online repositories. The names of the repository/repositories and accession number(s) can be found in the article/supplementary material.

## Author contributions

XF and ZS conceived the project and revised the draft. XF designed the methodology. AT and PG collected and cleaned the dataset. WP and RZ interpreted the results and drafted the manuscript. All authors have read and approved the final manuscript.

## Footnotes

1
https://www.genecards.org

2
https://string-db.org/

3
https://pubchem.ncbi.nlm.nih.gov/

4
http://www.rcsb.org/
